# Antimicrobial Activity of Lactoferrin-Related Peptides and Applications in Human and Veterinary Medicine

**DOI:** 10.3390/molecules21060752

**Published:** 2016-06-11

**Authors:** Natascia Bruni, Maria Teresa Capucchio, Elena Biasibetti, Enrica Pessione, Simona Cirrincione, Leonardo Giraudo, Antonio Corona, Franco Dosio

**Affiliations:** 1 Istituto Farmaceutico Candioli, Beinasco (To) 10092, Italy; natascia.bruni@candioli.it (N.B.); leonardo.giraudo@candioli.it (L.G.); 2 Department of Veterinary Sciences, University of Torino, Torino 10095, Italy; mariateresa.capucchio@unito.it (M.T.C.); elena.biasibetti@unito.it (E.B.); 3 Department of Life Sciences and Systems Biology, University of Torino, Torino 10123, Italy, enrica.pessione@unito.it (E.P.); simona.cirrincione@unito.it (S.C.); 4 Ambulatorio Veterinario Associato, Torino 10135, Italy; antonio@ambulatorioveterinario.eu; 5 Department of Drug Science and Technology, University of Torino, Torino 10125, Italy

**Keywords:** antimicrobial peptides, lactoferricin, milk proteins, food safety

## Abstract

Antimicrobial peptides (AMPs) represent a vast array of molecules produced by virtually all living organisms as natural barriers against infection. Among AMP sources, an interesting class regards the food-derived bioactive agents. The whey protein lactoferrin (Lf) is an iron-binding glycoprotein that plays a significant role in the innate immune system, and is considered as an important host defense molecule. In search for novel antimicrobial agents, Lf offers a new source with potential pharmaceutical applications. The Lf-derived peptides Lf(1–11), lactoferricin (Lfcin) and lactoferrampin exhibit interesting and more potent antimicrobial actions than intact protein. Particularly, Lfcin has demonstrated strong antibacterial, anti-fungal and antiparasitic activity with promising applications both in human and veterinary diseases (from ocular infections to osteo-articular, gastrointestinal and dermatological diseases).

## 1. Introduction

Antimicrobial peptides (AMPs) represent a vast array of molecules produced by virtually all living organisms as a natural barrier against infection. They are a primitive defence mechanism found in a wide range of eukaryotic organisms, throughout the taxonomic scale, including mammals, invertebrates and plants [1,2,3,4]. AMPs exhibit a broad range of activities against Gram-negative and Gram-positive bacteria, fungi, viruses, and parasites. To date, 2645 AMPs from various sources have been listed in “The Antimicrobial Peptide Database” [5], a database dedicated to natural AMPs. 

This rich source of antimicrobial agents has aroused growing interest, especially in the light of the decreasing effectiveness of antibiotics not only against severe infections, but also in treating common infectious diseases. Resistance to antibiotics has become a threat to global public health and is driving novel research into the development of new antimicrobial agents [6]. Innovation is thus needed not only for the development of new antibiotics (where, for example, the worldwide pipeline for new antibiotic classes active against highly resistant Gram-negative bacteria is almost dry) but also for combination therapies. By targeting different mechanisms of resistance simultaneously, combination therapy might help slow the emergence of resistance [7]. Moreover it has been strongly suggested that any synergy between these drugs and the immune response should be exploited in the treatment of bacterial infections [8].

Among AMP sources, an interesting class are the food-derived bioactive agents [9]. These AMP peptides together with other bioactive peptides are hidden inside food proteins (mainly milk proteins) and can be decrypted by proteolytic activity [10]. Dairy proteins, protein hydrolysates, and fermented dairy products have been shown to possess a wide spectrum of pharmacological activities: opioid, immunomodulatory, antimicrobial and antiviral, antithrombotic, growth-stimulating, and antihypertensive properties [11,12]. The class of AMP is particularly attracting and the “generally recognized as safe” (GRAS) status of food satisfies both consumers and industry. Research in the last twenty years has discovered several proteins and related peptides with interesting antimicrobial properties: milk proteins, such as β-lactoglobulin or α-lactalbumin and particularly lactoferrin (Lf) contain AMPs which have potential applications as pharmaceutical products [13]. The wealth of this research area is testified by specific conferences (e.g., XII International Conference on Lactoferrin) and a database named MilkAMP that contains all the physicochemical and microbiological characteristics of identified antimicrobial dairy peptides [14].

Recently, reports have demonstrated that the increased spectrum of activity of Lf and related peptides is larger than expected. Antiviral [12,15,16,17] and antiprotozoal activities [18] as well as tumor inhibition [19,20] and potent anti-inflammatory, anti-catabolic, and anti-oxidative effects [21] have been described. This article reviews the development of research on Lf-derived AMPs of bovine and human origin, and their applications, mainly as antimicrobial agents, for use by several administration routes and at different sites. Future prospects of lactoferrin-derived AMP will be also discussed. 

## 2. Lactoferrin: Distribution in Body Fluids and Clinical Efficacy

The whey protein lactoferrin (Lf) is an 80 KDa iron-binding glycoprotein that plays a significant role in the innate immune system, and is considered to be an important host defense molecule. A wide range of physiological functions such as antiviral, [22] antimicrobial, [23] antifungal, anti-parasitic [24], immunomodulatory [25] and antioxidant activities have been identified [26,27]. In humans, Lf is one of the major proteins in all exocrine secretions, such as colostrum (where Lf is found at the highest concentration, *i.e.*, 8 mg/mL), milk, tears, saliva, seminal and gastrointestinal fluids, nasal and bronchial mucosa, and plasma (0.2 µg/mL). Breast milk is the main source of Lf found in the gut of infants, and accounts for the initiation, development, and/or composition of the neonatal gut microbiota. Lf is also stored in the secondary granules of polymorphonuclear leukocytes and it has been reported that Lf regulates multiple signalling pathways to impart cytotoxic effects on cancer cell [19]. 

With regard to its antimicrobial effects, human Lf exhibits a very effective response against a wide range of bacteria, including species of *Streptococcus*, *Salmonella*, *Shigella*, *Staphylococcus*, and *Enterobacter* [28]. There is a vast literature describing the *in vitro* efficacy and animal-model benefits of Lf. The principal clinical results are reported below. 

One of the first applications of Lf in humans was in infant formula, and this has been the object of systematic and critical reviews [29,30]. The majority of studies have shown Lf to be safe in preterm/term new-born, and in older infants; in these children, protection against enteric and neonatal infections are the most likely biologically relevant activities of Lf. Several clinical trials are currently ongoing (data from www.clinicaltrials.gov).

Lf was also administered in a trial concerning *Giardia lamblia* infection; this is among the most common protozoan infection of the human intestine causing severe diarrhoea. A cohort of children with *Giardia* infection fed Lf showed a lower prevalence of colonization and better growth as compared to unfed children [31].

Lactoferrin, naturally present in the saliva, is associated with host defense against oral pathogens. The clinical usefulness of bovine Lf (bLf) combined with proteolytic enzymes to treat oral candidiasis or dry mouth has been examined [32,33] and a mucoadhesive tablet containing Lf has been developed [34]. Wakabayashi *et al.* evaluated the *in vitro* effects of Lf-related agents on the growth and biofilm formation of two periodontopathic bacteria, *Porphyromonas gingivalis* and *Prevotella intermedia*, which reside as biofilms in the subgingival plaque [35]. bLf used in combination with four antibiotics reduced the amount of a preformed biofilm of *P. gingivalis* compared with the reduction achieved with antibiotics alone. Improved liposomal formulations of Lf and lactoperoxidase significantly reduced the caries incidence in rat models [36]. These results demonstrate the potential usefulness of Lf for the prevention and treatment of periodontal diseases and as adjunct therapy in such diseases. 

Recently, Lf analysis was used as a sensitive assay for detecting the degree of periodontal inflammation and oxidative stress, and for monitoring the effects of periodontal therapy as well [37]. The Lf level was higher in the periodontitis group of patients compared to the healthy group, and the measured level decreased in the former group after periodontal therapy. Higher Lf levels were associated with higher values of clinical parameters (such as gingival index, plaque index, probing pocket depth, clinical attachment level) both before and after therapy. This overall research indicates that Lf plays an important role during periodontal disease, and crevicular Lf quantification could be a marker for detecting periodontal inflammation, oxidative stress, and monitoring periodontal therapy.

The role of fermented milk and whey proteins in controlled clinical trials regarding *Helicobacter pylori* infections was recently reviewed by Sachdeva [38]. Although the available evidence suggests that bLf is beneficial for *H. pylori* eradication (Recommendation Grade-A), unlike AMP derived from Lf [39], the extent of the documented benefit is small, and deserves further exploration. Other clinical trials on Lf are related to: (i) the prevention of hospital-acquired infections; (ii) the treatment of cystic fibrosis [40] in combination with hypothiocyanite; (iii) clinical efficacy against the common cold in combination with the IgG-rich fraction of whey proteins [41].

Regarding potential Lf side effects, advanced clinical trials, including the treatment for *H. pylori* infection [42], iron deficiency anemia in pregnant women [43] and new-born sepsis [44] did not report any significant adverse effects or intolerance.

**Figure 1:**
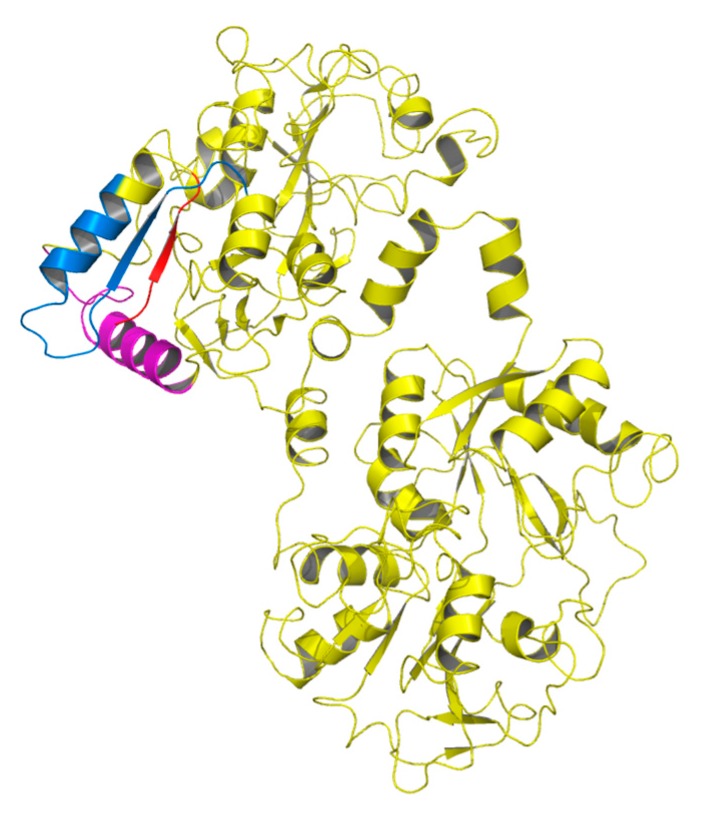
Overall structure of lactoferrin showing positions of the functional peptides Lf(1–11) (red), lactoferrampin (pink), and lactoferricin (blue) in the N-terminal lobe.

A number of functional peptides are produced from Lf by the action of proteolytic enzymes, and many Lf-derived antimicrobial peptides have been isolated and characterized (Figure 1); three have been studied in some detail [45]. They originate from the N-lobe of Lf, and their antimicrobial activity is chiefly linked to hydrophobicity, cationic charge, and helical conformation, which render these peptides amphiphilic molecules. Most of them cause membrane depolarization (like the antibiotics colistin and polimixin B) [46]. However, complex mechanisms, such as inhibition of the synthesis of macromolecules [47], and synergic action with host innate immunity compounds were also described [48]. The three peptides are Lf(1–11), lactoferricin (Lfcin) and lactoferrampin (LFampin); the alphabetic letter indicates the species of origin, e.g., (b) for bovine and (h) for human [49].

## 3. Lf Derived Peptides: Lf(1–11)

**Figure 2:**
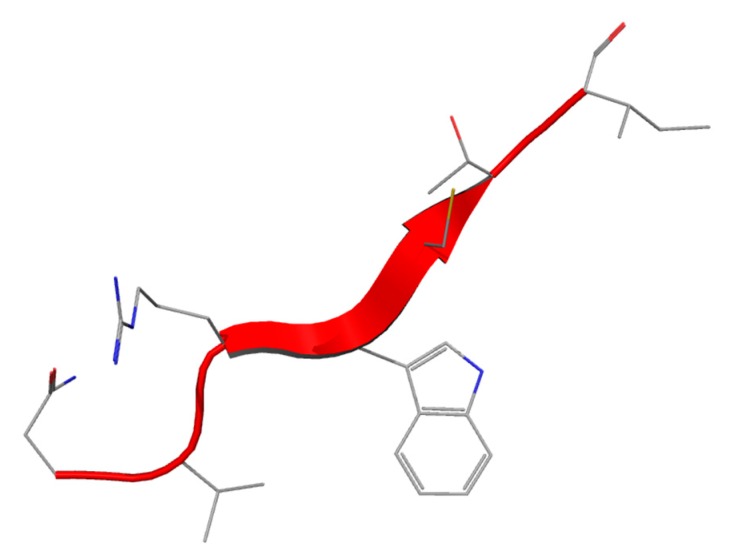
Structure of Lf(1–11) peptide.

Lf(1–11) is the oligopeptide including the first eleven aminoacid residues of the Lf molecule (Figure 2). The sequence comparison of Lf(1–11) from six mammalian species (Table 1) shows that some important features such as the highly cationic nature of the peptide is maintained (pI > 11, ranging from 11.70 (bovine) to 12.5 (human)) and the hydrophobic residues valine and tryptophan V6 and W8 are conserved in all species. Investigation of the structural and dynamic properties in water and membrane-mimicking solvents show that hLf(1–11) effectively interacts with the membrane, whereas the control peptide (a scrambled analogue) did not show such conformation [40].

### 3.1. In Vitro Antibacterial and Anti-Fungal Activity of Lf(1–11)

In a recent study, hLf(1–11) (milkAMP database id = LFH0004) was investigated for its ability to treat infections caused by multi-drug-resistant bacteria. The results showed that hLf(1–11) was active against various Gram positive bacteria such as *Staphylococcus* spp. (including MRSA), and *Streptococcus mitis* (MIC values ranged from 1.6 to 6.3 µg/mL) as well as Gram-negative bacteria: *Acinetobacter baumannii*, *Pseudomonas* spp., *Klebsiella* spp. and *E. coli* (MIC values 6.3 to 12.5 µg/mL). As far as Yeasts are concerned in *Candida* spp. (MIC > 12.5 µg/mL) were found [49].

This peptide’s anti-fungal action mechanism appears to involve a particular sequence of events. The peptide interacts with structural elements of the plasma membrane of the blastoconidia, and is taken up in an energy-dependent manner. Inside the cell it triggers the energized mitochondria to synthesize and secrete ATP, and the peptide is released extracellularly, where it interacts with surface ATP binding sites, resulting in pore formation. These events induce progression towards cell death, possibly involving mitochondria [50]. Another study demonstrated the involvement of fungal endogenous thiols and reactive oxygen species (ROS) in the candidacidal activity exerted by hLf(1–11). In this study, hLf(1–11) caused a decrease in the internal thiol levels of *Candida albicans* by 20% and an increase in the level of its production of ROS in a dose-dependent manner; a correlation between ROS production and candidacidal activity was also found [51].

Little has been reported concerning the synergistic effects of hLf(1–11) with antibiotics. It has been observed that Lf or hLf(1–11), added at sub-inhibitory concentrations to antifungal agents such as clotrimazole, ketoconazole, fluconazole, or itraconazole, reduces the minimum inhibitory concentrations of these agents against *Candida* species [52]. The peptide was highly active against fluconazole-resistant *Candida albicans* at non-candidacidal concentrations, acted synergistically with fluconazole against this yeast and a fluconazole-sensitive *C. albicans* strain, as well as against *C. glabrata*, *C. krusei, C. parapsilosis* and *C. tropicalis*. When these yeasts were exposed to hLf(1–11) for five minutes and then incubated with fluconazole, they were effectively killed, while no candidacidal activity was observed when they were incubated first with fluconazole and then exposed to the peptide: this shows that the candidacidal activity is initiated by the peptide, whereas fluconazole is only required during the effector phase [53].

### 3.2. Main in Vivo Results in Human and Animal Models

hLf(1–11) showed activity with 2.5 log reduction against an antibiotic-resistant *S. aureus* strain (at 12 μM) [54]; it retained its activity when bound to bone cements used clinically [55]. In rabbit model trials using cement containing hLf(1–11), two studies [56,57] revealed a significant reduction of bone-injected *S. aureus* ATCC 10832 bacteria or *S. aureus* (W234) compared with the control group. The results clearly showed that hLf(1–11) has the ability to reduce osteomyelitis, with microbiological results similar to those of gentamicin [57]. In an earlier study, the intravenous injection of hLf(1–11) at 0.1 or 1 nmol successfully reduced murine muscle infections (1 × 10^7^ CFU) caused by the methicillin-resistant *S. aureus* strain 2141 (MRSA), *Klebsiella pneumoniae* ATCC 43816 [54], *S. aureus* 25923 [58], and various multidrug-resistant *Acinetobacter baumannii* [59]. 

In an interesting report using labelled hLf(1–11), the administration routes of the peptide were compared (oral, i.p., i.v. and s.c.) on mice infected with multidrug resistant *S. aureus* [60]. A dose-dependent effect was observed with increasing i.v. doses, from 0.04 to 40 mg/kg of body weight, with maximal reduction of viable bacteria in infected thigh muscle of 4 log CFU/g of tissue compared with control. hLf(1–11) was rapidly removed from the circulation after i.v. administration (blood half-life t = 5–10 min) through the kidneys. This administration route produced the highest concentration in muscle tissue (0.9% ID/g). Removal was slower for orally-administered peptide, and 38% ID/g of the radioactivity still remained in the stomach and 40% in the intestine 2 h after administration [60].

Following the promising *in vitro* antifungal results, *in vivo* activity of hLf(1–11) against fluconazole-resistant *C. albicans* was investigated using neutrocytopenic mice infected with *C. albicans* Y01-19. A reduction of clinical signs and symptoms of the infection was observed at a dose 0.4 μg/kg of body weight, much lower than that found in *in vitro* experiments [49]. The most likely explanation for the levelling off of the antifungal effects of hLf(1–11) is that the peptide induces multiple processes that contribute differently to its antifungal activity. Furthermore, administering hLf(1–11) at up to 10,000 times the therapeutic dose produced no significant adverse or toxic effects, and consequently the therapeutic window of the peptide is very wide.

The safety of Lf(1–11) was investigated during the first three trials conducted in humans using ascending doses of the peptide. The investigation was done in healthy volunteers and to patients receiving autologous haematopoietic stem-cell transplantation following conditioning with high-dose melphalan for multiple myeloma or lymphoplasmocytic lymphoma. Intravenous doses of up to 5 mg daily for 5 days showed a very favourable side-effect profile. The only undesirable effect was an elevation of transaminases, which may be due to hLf(1–11), although current data does not allow any cause-effect relationship to be postulated [61].

The safety and tolerability of hLF(1–11) is under investigation in some clinical studies, sponsored by AM-Pharma, but currently no other recruiting studies are on-going (from www.clinicaltrials.org database) thus the future of pharmaceutical applications appears unclear. 

## 4. Lf-Derived Peptides: Lactoferricin

**Figure 3:**
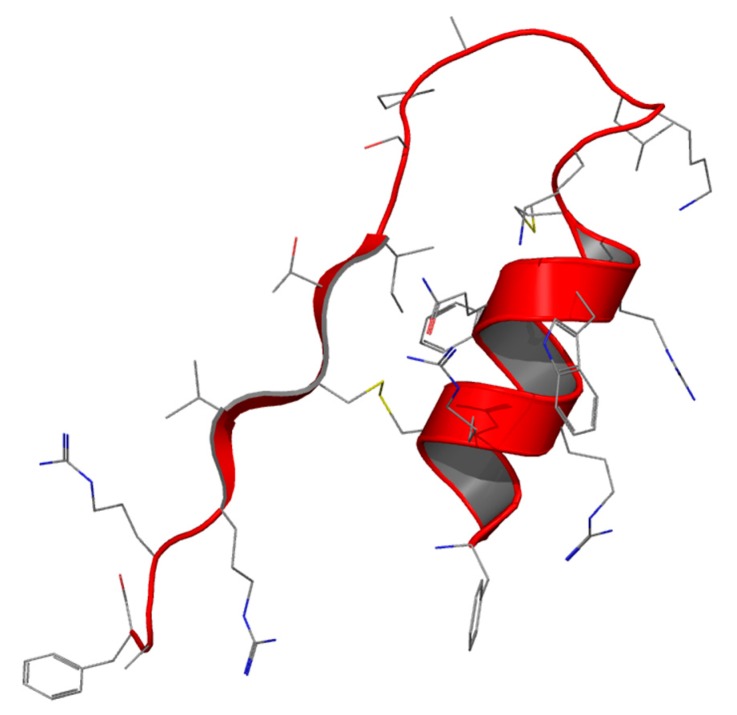
Structure of Lfcin peptide.

After Lf proteolysis by pepsin under acidic conditions, a 25-residue peptide (Lf amino acid residues 17–41) lactoferricin (Lfcin) was identified [62] The peptide has an abundance of basic amino acids including lysine and arginine, as well as hydrophobic residues like tryptophan and phenylalanine. In all Lfcins (derived from different mammalian species) a loop region with an intramolecular disulfide bridge is present (milkAMP database id = LFB0084) (Figure 3) [62]. Table 2 shows the structure of Lfcin of different origins [63]. The current hLfcin sequence contains this loop, but in this case a second disulfide bond extends the overall structure, which is about twice as long as bLfcin. hLfcin is composed of two fragments corresponding to 1–11 and 12–47 connected by a disulfide bridge [62,64] (milkAMP database id = LFH0009). 

Both bLfcin and hLfcin exist as an amphipathic α-helix in Lf but, after pepsin digestion, the former is transformed into amphipathic β-sheet hairpin in an aqueous environment, whereas the latter (bulkier model) also possess a parallel β-sheet that is lost after digestion, preserving the α-helix [64]. The retention of the α-helical region in hLfcin may be directly related to the additional length of the peptide. The ability of Lfcin to form amphipathic structures with net hydrophobic and positively-charged faces (Table 2) is a trait shared with other peptides having antimicrobial activity [65].

### 4.1. In Vitro Antibacterial and Anti-Fungal Activity

The first study, in 1992, on Lfcin as an AMP reported it to be a more potent antibacterial and anti-fungal agent than intact Lf; it was shown to cause a rapid loss of colony-forming capacity in most of its targets [66]. Comparing the activity of Lfcin from different species (cow, mouse and goat) showed that bLfcin was the most potent [63]. For example, the MIC of bLfcin against certain E. coli strains was found to be around 30 μg/mL whereas a MIC of with 100 μg/mL was detected for hLfcin. bLfcin showed bactericidal activity against a range of Gram-positive and Gram-negative bacteria [67], whereas hLfcin only exerted bacteriostatic activity [62]. The wide range of activities are shown in Table 3; the MIC as antibacterial, antifungal and anti-protozoarian activities are taken from the milkAMP database [14].

Very recently, data have been reported on Lfcin and other Lf peptides derived from Lf active against *Mycobacterium avium* [68]. Both human and bovine Lfcins, as well as Lf(1–11), were active against *M. avium* strains of different virulence, the bovine peptide being more active than its human counterpart. However, some strains, such as *Pseudomonas fluorescens*, *Enterococcus faecalis*, and *Bifidobacterium bifidum*, were found to be resistant to Lfcin [69].

The antibacterial activity of Lfcin is thought to involve the disordering and alteration of the permeability of the bacterial membrane, resulting in inhibition of macromolecular biosynthesis and ultimately cell death [70]. In terms of the structure-activity relationship, the mechanism of action of Lfcin has been attributed to the 11-amino-acid amphipathic α-helical region [71], and the importance of the initial electrostatic interaction is highlighted by the high overall positive charge of these peptides; a net charge of at least +4 is necessary for optimal antibacterial activity [72]. This is confirmed by the fact that murine Lfcin (mLfcin), which contains two glutamine residues, lacks antibacterial activity (Table 2) [63] and also by the increased activity of C-terminally amidated undecapeptides derived from various Lfcins [72]. Since arginine can interact both electrostatically and through multiple hydrogen bonds with the negatively-charged surface of bacteria, it is thought that this amino acid is the most effective for targeting the peptide to the bacterial membrane. In addition, the guanidinium group adds bulk to the side chain, thereby potentially contributing to membrane disruption [71]. Once the positively charged residues bring Lfcin into contact with the bacterial cell, the hydrophobic residues (in particular tryptophan) interact with the lipophilic portion of the membrane, becoming embedded in its surface and destabilizing the packing of the phospholipids. At least two tryptophan residues (the best value being with three residues) are required to ensure a maximal thinning of the membrane in a certain radius around the peptide [73].

On Gram-negative bacteria, antimicrobial peptides act on lipopolysaccharides, whereas on Gram-positive bacteria Lfcin acts on lipoteichoic and teichoic acids. Studies have indicated that bLfcin leads to depolarization of the cell membrane without causing the lysis of the cells [70], that it exerts its bactericidal effect initially by acting on the bacterial cell surface, and subsequently on the cytoplasmic contents [74].

The role of disulfide is not yet fully understood, and Liu *et al.* [74] recently failed to find a significant difference in MICs between the disulfide-bridged peptide and its linear counterpart, while another study showed three-times-higher activity for the disulfide-bridged peptide [63]. Intracellular targets, such as phosphoenolpyruvate carboxylase, were detected using *E. coli* proteome chips, indicating that one of Lfcin’s mechanisms of action may be associated with pyruvate metabolism [75]. Furthermore, bLfcin inhibits phosphorylation of the response regulators and of the two-component system’s (TCSs) cognate sensor kinases. The role of the TCS is to protect the integrity of bacterial cell membranes against antimicrobial peptides [76]. However, the homologous examination of response regulators and sensor kinases in probiotics, including *Bifidobacterium* and *Lactobacillus*, showed that this motif was not present, suggesting that bLfcin may have only marginal effects on the microbioma probiotics in the intestine [76].

In addition to antimicrobial properties, Lfcin of human and bovine origin has also been found to be effective in inhibiting the classical complement pathway. Both Lf and Lfcin increase interleukin-8 release from polymorphonucleate leukocytes, suggesting their immunomodulatory function [77]. This implies a role of these peptides in suppressing the inflammatory effects caused by bacteria [78]. Lfcin was found to be produced in the human stomach, indicating that this peptide is generated *in vivo* for host defense [79].

In regard to its toxicity on eukaryotic cells, measured as haemolytic activity, bLfcin displayed slight hemolytic activity at a concentration of 64 µg/mL, exhibiting a marked antimicrobial activity against most of the test bacteria [74]. However, a study of the relationship between structure and activity found that the undecapeptide structure is essentially non-haemolytic, but that undecapeptides containing more than three tryptophan residues produced 50% haemolysis of human red blood cells at concentrations above 400 μg/mL (>230 μM) [72]. Toxicity seems also related to increased hydrophobicity. In a strategy to improve antimicrobial activity of Lfcin peptides were linked by *N*-acylation to hydrophobic chains. The derivatives resulted in higher antibacterial activity but also in higher toxicity towards eukaryotic cells [80,81].

Apart from having a broad antibacterial spectrum, bLfcin was also found to be efficacious against yeasts, such as *Candida albicans*, *Cryptococcus uniguttulatus*, *C. curvatus*, *C. albidus* and *Trichosporon cutaneum* [67,82] (see Table 3). The anti-fungal mechanism has not yet been fully clarified. bLfcin interacts with and disrupts the integrity of the *Candida* spp. membrane within the range 18 to 60 µg/mL (MIC). Cells of nine different strains exposed to bLfcin exhibited severe ultra-structural damage, reflecting the peptide’s induction of an autolytic response [83].

### 4.2. Synergy with Antibacterial and Anti-Fungal Drugs

Synergistic effects, deriving from the combination of active agents with different modes of action, provide an attractive therapeutic option (see Table 4). The loss of inner membrane integrity may promote the uptake of other agents, for example antibiotics or other antibacterial peptides, leading to synergy with conventional antibiotics. In this respect, Lfcins also appear to affect the cytoplasm physiology of fungal cells. In addition, some azole-resistant *C. albicans* strains are inhibited by fluconazole or itraconazole to a greater extent in the presence of relatively low concentrations of Lf or bLfcin. Conversely, no cooperative effect with non-azole types of antifungal agents, such as amphotericin B or 5-flucytosine, was observed. These findings indicate that LF or bLFcin may play a valuable role in inhibiting the mycelial form of azole-resistant *C. albicans* [52,84].

Based on Lfcin’s high antibacterial and antifungal activity, in order to identify possible synergic therapeutic strategies, Lf peptides have been tested in association with drugs. Following initial evaluations, Vorland *et al.* examined whether bLfcin interfered with the action of various antibiotics against *E. coli* and *S. aureus* [85]. For what regards *E. coli*, marked synergism was observed with erythromycin, partial synergism with penicillin G, vancomycin, and gentamicin, whereas there was no effect with cycloserine or colistin. As far as *S. aureus* is concerned, only penicillin G acted in partial synergism with bLfcin, whereas bLfcin antagonized vancomycin and gentamicin also at low concentrations. The difference in activity is related to the different mechanisms of action of antibiotics on both the membranes and the intracellular targets. In some cases, however, bLfcin may facilitate the uptake of antibiotics across the cell envelope.

Longhi *et al.* analysed the susceptibility to fluoroquinolones of uropathogenic *E. coli* strains, and the influence of bLfcin on the activity of norfloxacin and ciprofloxacin against these strains [87]. The research revealed synergistic, partial synergistic, or indifferent effects depending on the tested *E. coli* strains. Furthermore these results demonstrated that the association of fluoroquinolones with bLfcin could allow the use of these therapeutic agents at lower concentrations for a reasonable number of *E. coli* strains.

Liu *et al.* found a synergistic effect between bLfcin and aureomycin and cecropin A, on *E. coli*. No synergy, but rather independent action, was found in all bacteria tested when combining bLfcin and neomycin. No antagonism was observed between the antibacterial agents used and bLfcin against *E. coli*, *S. aureus* and *P. aeruginosa*. The MICs for the antibiotics markedly decreased when the peptide was added, as expected, indicating that bLfcin might act in combination with these antimicrobial agents against *E. coli*, *S. aureus* and *P. aeruginosa* [74].

Wakabayashi investigated the effects of bLfcin combined with antibiotics or various other compounds against *S. aureus* [88]. Among conventional antibiotics, minocycline increased the bactericidal activity of bLfcin against *S. aureus*, but methicillin, ceftizoxime, and sulfamethoxazole-trimethoprim did not have such an effect. The combination of minocycline and bLfcin had synergistic effects against three antibiotic-resistant strains of *S. aureus*. Further, 33 compounds were screened, including acids and salts, alcohols, sugars, lipids, amino acids, proteins and peptides, were tested in combination with bLfcin and among these medium-chain (8, 12, 14 carbons) monoacylglycerols increased the bactericidal activity of bLfcin against three *S. aureus* strains.

A cooperative action of milk proteins was investigated by Lopez-Esposito [90]. In this study Lf, bLfcin, and alphaS2-casein were assayed against *E. coli*, *S. epidermidis*, *Listeria monocytogenes* and *Salmonella cholerae-suis*, in combination with nisin. This bacteriocin produced by *Lactocococcus lactis* spp. *lactis* is primarily active against Gram-positive bacteria, and has found practical application as a food preservative. The synergy was observed for the combination Lf and bLfcin against *E. coli* and *S. epidermidis*. When bLfcin was combined with nisin, an antagonistic effect was found against *E. coli*, whereas the synergy index achieved against *S. epidermidis* revealed an additive interaction. Murata *et al.* [92] subsequently identified a milk RNAse protein (15 kDa) able to double or quadruple the antimicrobial activity of bovine Lf and LFcin against G-negative and positive bacteria. 

### 4.3. Anti-Parasitic Activities of Lfcin

A synergistic effect against *Entamoeba histolytica*, was found by Leon-Sicairos *et al.* between metronidazole and Lf or bLfcin: in the presence of 31 µM of Lf or 323 µM of bLfcin, approximately one-third to one-fifth of the full metronidazole concentration effectively killed the majority of amoebas. This result opens promising perspectives for reducing the dose of antiamoebic drug in patients [89].

The ascertained giardicidal activity of bLf has the limit to be hampered by metal ions. It has been demonstrated that the most efficient activity against *Giardia*, is due to bLfcin. The latter also has the advantage, as compared to bLf to be insensitive to the inhibiting action of metals, except for Fe^3+^ [93]. The study authors opined that, as the intestinal lumen has little free iron, it is likely that Lf-derived peptides remain active within the human small intestine.

Treatment of *Toxoplasma gondii* with bLfcin leads to inactivation of the parasite’s ability to penetrate the host cell [94]. This protective effect in mice was confirmed by Isamida *et al.* [95] using oral or i.p. administration of the peptide (5 mg and 0.1 mg respectively). Subsequently, Omata *et al.* observed that sporozoites of *T. gondii* and *Eimeria stiedai* pre-incubated with Lfcin showed decreased penetration activity of mouse embryonal cells; mice inoculated with 105 sporozoites preincubated with Lfcin showed a higher survival rate than those inoculated with the same number of untreated sporozoites [18].

### 4.4. Preclinical and Clinical Trials of Lfcin

On the basis of *in vitro* data confirming bLfcin as the best performing peptide, with broad-range activities and low MIC values, several preclinical trials were run; the principal results are reported below, classified by disease treated or by administration route. 

#### 4.4.1. Ocular Infections

Recently, Oo *et al.* [86] reported that two antibiotics (ciprofloxacin and ceftazidime) act synergistically with bLfcin against various strains of *P. aeruginosa* isolated from corneal infections *in vitro*. In mouse corneas, the addition of bLfcin as adjuvant enhanced the activity of ciprofloxacin, with 1.5 log reduction of bacterial growth compared with negative control, although the peptide alone had no significant effect. Moreover, bLfcin with or without ciprofloxacin appeared able to reduce myeloperoxidase activity and consequently host inflammatory response *in vivo*.

More recently, an *ex vivo* study on the efficacy of the combination of bLfcin with other antifungal agents was performed. The aim was to assess the performance of such a combination in the inhibition of evaluated of biofilm formation of three fungal strains, *Aspergillus fumigatus*, *Fusarium solani*, and *Candida albicans*, isolated from patients with keratitis [96]. Biofilm eradication is now a major challenge, to overcome the incidence of drug resistance in keratitis treatment. This disease constitutes one of the most important causes of ocular morbidity and sight loss in developing nations, as well as in contact lens users, as reported in recent epidemics of *Fusarium* spp. keratitis. bLfcin, in combination with antifungal agents, increased the susceptibility to fluconazole, voriconazole, and amphotericin B and, when added to a lens-care solution, drastically reduced the mature biofilm on contact lenses. This finding suggested that bLfcin might be a promising candidate for clinical use in improving the susceptibility of biofilm to antifungals, and could also be used as an antibiofilm-antifungal additive in lens-care solutions.

#### 4.4.2. Osteo-Articular Diseases

In addition to antibacterial and antifungal uses, bLfcin’s anti-catabolic and anti-inflammatory effects were recently assessed in *ex vivo* experiments on human articular cartilage and synovium [97]. bLfcin demonstrated chondroprotective properties, damping the inflammatory response in synovial fibroblasts, and may thus be seen as a promising molecule in the prevention and/or treatment of degenerative joint diseases. Additionally, considering its antimicrobial activities, bLfcin may also bring therapeutic benefits to septic arthritis. 

#### 4.4.3. Gastro-Intestinal Diseases

The first reports of the bacteriostatic effects of Lf and Lf pepsine-hydrolysate (LFH) after per os administration, concerned *Enterobacteriaceae* in the gut environment [98], The same author demonstrated the efficacy of bLf and LFH to control and limit the growth of different strains of *Clostridium* spp. in a mouse model [99].

#### 4.4.4. Dermatological Diseases

Lf and bLfcin have been tested as possible therapeutic agents for the treatment of dermatophytosis, one of the most common infectious diseases of the stratum corneum. The *in vitro* MIC of Lf and Lfcin against *Trichophyton* spp. demonstrated a great variability, depending on the strain tested and medium used, in contrast of what observed with the antifungal drug griseofulvin which gave constant and reproducible results. The efficacy of oral Lf administration was assessed on guinea pigs experimentally infected with *Tinea corporis* and *T. pedis*. The study authors assumed that Lf did not have any direct antifungal activity, but that it enhanced the inflammatory response involving cell-mediated immunity required for the cure of dermatophytosis [100].

### 4.5. Veterinary Applications

Mastitis is one of the most significant and costly diseases in dairy cattle. It is an intramammary bacterial infections caused by several bacteria, with *S. aureus*, *Streptococcus uberis*, and *Streptococcus dysgalactiae* being the most frequently involved pathogens. Intramammary treatment with Lf was not satisfactory for overcoming beta-lactam resistant *S. aureus* infection. However, Lf co-administered with penicillin G increased the cure rate (from 12.5% to 33%), reducing beta-lactamase activity in resistant *S. aureus* strains [101]. The strong activity against mastitis pathogens of AMP and bLfcin, in particular, has spurred interest in their potential application to the control of udder infections. In an *in vivo* trial, Kawai *et al.* tested an infusion of LFH in cows affected by subclinical mastitis, caused by various bacteria, including *E. coli* and staphylococci [102]. The results showed a significant reduction of bacteria in the mammary tissue already on day one after infusion, and eradication of the disease after 14 days. Recently, bLfcin exhibited *in vitro* biocidal activity against both the algae *Prototheca zopfii*, the causative agent of protothecal mastitis, and several yeasts causing fungal mastitis isolated from clinical cases of bovine mastitis However these promising *in vitro* findings must be confirmed *in vivo* [103]. In a different approach, bLfcin was expressed in goat mammary gland, using a plasmid vector as preventive therapy [104]. The results confirmed the persistence of the peptide in goat’s milk for 6 days after injection of the recombinant plasmid, but with variations in the concentration after 3 days. Milk produced during this period was active *in vitro* against *S. aureus* ATCC 25923 and *E. coli* D12K31.

hLf and Lfcin have also been studied as components of dietary supplementation in livestock, and particularly in pigs. Tang *et al.* analysed the role of Lfcin to replace colistin sulphate in a study on piglets weaned at 21 days of age and challenged with enterotoxigenic *E. coli*, that examined gut microflora, circulating cytokines, and intestinal mucosal morphology [105]. When cipB-Lfcin, a fusion protein that releases Lfcin in the animal stomach, was given as a dietary supplement at the dose of 100 mg/kg, the development of villus-crypt architecture of the intestinal mucosa was observed. Because Lfcin can decrease the concentration of *E. coli* and keep the gut tissue healthy, pigs fed with cipB-Lfcin had lower serum levels of circulating cytokines than pigs fed a standard or control diet [95]. Further studies of the use of these peptides as an alternative to antimicrobial growth promoters in pig production comprised construct cipB-Lfcin-Lframpin [106]. Piglets fed with construct (100 mg/kg) or control diets were challenged with enterotoxigenic *E. coli*. The AMP diet enhanced growth performance to a similar extent as did colistin sulphate. Analogous results were obtained using fusion protein containing bLfcin or Lfampin produced by a recombinant of the yeast *Pichia pastoris* [107,108].

A further study investigated the effects of bLfcin on performance, faecal score, and dry matter of weaned piglets orally challenged with enterotoxigenic *E. coli* F4, confirming bLfcin’s activity combined with its absence of toxicity [109]. 

Recently, an *in vivo* study comprised treating dogs affected by external otitis with bacterial and yeast overgrowth, using an emulsion containing bLfcin, verbascoside and glycerophosphoinositol lysine [110]. After 7 days’ treatment, there was a significant decrease in microbial overgrowth, together with a clinical improvement in the otitis, suggesting the therapeutic combination possessed synergistic antibacterial and antifungal activity. An *in vitro* study in the present authors’ laboratory confirmed the anti-fungal activity of bLfcin against *Malassezia* spp. isolated from dogs or cats affected by fungal otitis (unpublished data). 

### 4.6. Lfcin for Food Applications

On the basis of its wide spectrum of activity, bLfcin is also a promising candidate as preservative in various foods and beverages. The main applications of AMP are well summarized in a recent review [13]. Chiefly considering bLfcin and LFH, the principal studies examined were as follows:

#### 4.6.1. Dairy products

Quintieri and co-workers [111] evaluated the effect of bLfcin to control spoilage by mesophilic bacteria in Mozzarella cheese and, more recently, that of LFH in controlling *P. fluorescens*, responsible for cheese pigmentation in high-moisture Mozzarella cheese. LFH (rich in bLfcin) efficiently counteracted the chromatic spoilage of cheese throughout the storage time [112]. The inclusion of LFH in the holding liquid did not significantly affect the viable cell counts of useful microorganisms; however, resistance of AMPs to proteases becomes a major concern, because they rapidly degrade in food matrices, especially those containing lactic acid bacteria whose proteolytic attitude is very strong. This aspect strongly limits their action time. For example, bLfcin was reported to have a short half-life in contact with food microbial communities [111,112]. For these reasons, bLfcin resistance to microbial proteolysis was also assessed using different strains of yogurt starter (*Streptococcus thermophilus* and *Lactobacillus delbrueckii* sp. *bulgaricus* strains). However in acidic conditions (pH 4.5) the bLfcin resistance to hydrolysis was confirmed [113].

#### 4.6.2. Oenological applications

Recently, the use of both bLf and bLfcin was examined, in a study that assessed their control over food spoilage bacteria and yeasts. Enrique *et al.* [114] evaluated both *in vitro*, and subsequently in micro-vinification experiments, the antimicrobial activity exerted by LFH and one synthetic peptide derived from bLf (bLfcin17e31) against wine-related Gram-positive spoilage bacteria. The study authors demonstrated the additional killing effect of bLfcin17e31 on bacterial cells belonging to *Pediococcus damnosus* and *Oenococcus oeni*, already stressed by changes in chemical composition of the must caused by *Saccharomyces cerevisiae* fermentation.

#### 4.6.3. Fruits and vegetables

Recently, both bLf and bLfcin were investigated for their potential in controlling spoilage pathogens in fruits and vegetables. Tests carried out on mandarins with bLfcin and Lf (17–31) showed a significant protection against *Penicillium digitatum* at a concentration close to the *in vitro* MIC value [115]. More recently Baruzzi *et al.* demonstrated the interesting activity of bLfcin in controlling microbial spoilage in ready-to-eat leafy vegetables during cold storage [116]. These studies introduce alternative approaches to fungicides in fruit and vegetable production. 

#### 4.6.4. Meat products

bLfcin is currently also being assessed to control food-borne pathogens in meat matrices. Initial studies on the addition of bLfcin to ground meat inoculated with enterohemorrhagic *E. coli* O157:H7 did not significantly reduce the bacterial population [117], although LFH exerted antimicrobial action on this strain in controlled experiments [118]. Similar results were obtained on beef plates and adipose tissue after treatment by immersion in a solution of 10 μg/mL of bLfcin [119]. The possibility of combining LfcinB and high pressure to reduce colonization of chicken fillets preinoculated with *Pseudomonas fluorescens* ATCC948, *Listeria monocytogenes* CECT5725 and *E. coli* O157:H7 strain CECT 4972 was evaluated, although if failed to produce a relevant reduction [120,121]. In another approach, LFH was applied to ground beef and meat fractions [122]. In conclusion of this chapter it is worth highlighting that the strategy of employing milk-derived AMPs to control spoilage bacteria and food-borne infections not only satisfies the need to reduce the use of antibiotics and food preservatives but also allows to reduce the use of salt and sugars with benefits for consumer’s health.

## 5. Lf Derived Peptides: Lactoferrampin

**Figure 4:**
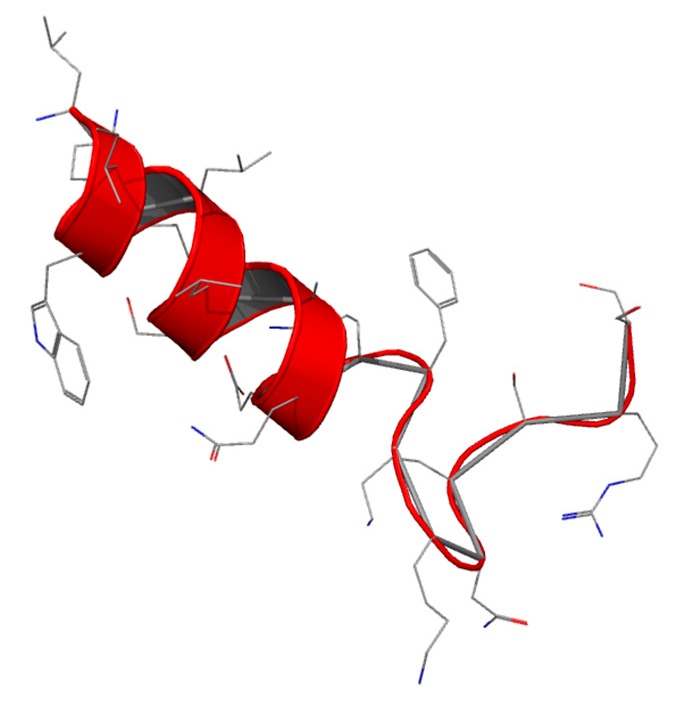
Structure of Lfampin.

Lactoferrampin (Lfampin) has recently been identified as being of potential interest by sequence scanning, based on the common feature of antimicrobial peptides: a highly positive charge, a hydrophobic domain and hence an amphipathic character (Figure 4).

Lfampin comprises residues 268–284 in the N1 domain of Lf, and was found to be located in close proximity to Lfcin. However, the bactericidal activity of LFampin differs from that of bLfcin, although both peptides share amphipathic and cationic features; they have a markedly different amino acid composition and chain length, and therefore their structures differ greatly. The sequence comparison of Lfampin from six different species shows a uniform preponderance of cationic amino acid residues among hydrophobic residues (Table 5). As an antimicrobial peptide Lfampin plays a key role in membrane-mediated activities of Lf [123]. Following the initial discovery of LFampin 268–284 (produced by solid-phase chemistry), a slightly longer sequence (produced by action of a single endopeptidase on bLf) was published that included an additional N-terminal helix cap region, Asp-Leu-Ile. It was found that the longer peptide LFampin 265–284 had higher activity [123]. For both peptides, the helical conformation was found to be critical for effectiveness against Gram-positive bacteria [124].

The sequence comparison of Lfampin from six different species shows a uniform preponderance of positively-charged residues among the hydrophobic domain containing tryptophan, a residue that is involved in membrane insertion.

Lfampin exhibits broad antimicrobial action against several Gram-positive and Gram-negative bacteria, yeast and parasites: data taken from the MilkAMP database are reported in Table 6 [14,123].

Although no advanced preclinical trials or applications have yet been reported, the study by Flores *et al.* shows that the combination of ampicillin with LFcin 17–30 or LFampin 265–284 enhanced the inhibitory effect on growth of *S. aureus* (99.9%) for both peptides, suggesting that a synergistic effect occurs. These data strongly suggest that LFcin 17–30 and LFampin 265–284 act synergistically with antibiotics against multi drug resistant *S. aureus* strains *in vitro* [91].

## 6. Conclusions

With the steady increase in the number of multidrug-resistant pathogens, many patients are looking to alternative medicine instead of classical antibiotics and antimicrobial agents. It has thus become necessary to explore natural resources for new, alternative and/or complementary medicines. In this search for novel antimicrobial agents for the future, Lf, a multifunctional protein that participates in a range of important physiological processes, offers a new source with potential pharmaceutical applications. Moreover, Lf fragments or derivatives are still being explored, through both chemical synthesis and tryptic digestion, as has recently been described [125].

The best demonstrated effects of Lf and Lf AMP in the treatment of various infectious diseases caused by bacteria, fungi, and protozoa in humans and animals have been described; however, considerable research remains to be done to achieve a better understanding of Lf and Lf-derived AMPs activity. Actually, the functional peptides till now produced from Lf and studied, Lf(1–11), Lfcin and LFampin, are still underexplored but promising results begin to be obtained suggesting new interesting antimicrobial actions that can be exploited. 

Lf(1–11) is active *in vitro* and *in vivo* against various bacteria and yeast although showing few synergistic effects with antibiotics. Lfcin displays a higher efficacy potent as an antibacterial, anti-fungal and antiparasitic agent than intact Lf, particularly in ocular infections, osteo-articular, gastrointestinal and dermatological diseases. bLfcin is the most potent Lfcin, and is also used in veterinary medicine. Bovine mastitis, intestinal infections in piglets, and canine dermatitis are the most important *in vivo* applications. 

Both bLf and bLfcin have recently been tested for food applications due to their significant antibacterial and antifungal activities, combined with a wide safety profile. They find interesting applications in food preservation from both spoilage and pathogenic bacteria and fungi. This strategy may allow to reduce the use of chemical preservatives, reducing food loss due to spoilage, and lead to the development of “novel” food products (with lower salt content, less acidic, *etc*.) able to satisfy the demands of consumers and industry.

Further, the ascertained protective roles of bLfcin, which have been demonstrated against pathogens, and inflammation, but also cancer, make the molecule a centerpiece for the development and application of drug candidates and functional foods. Natural origin, safety, marked activity, and possible use on the industrial scale, are the main features that make Lfcin interesting and promising as a therapeutic agent. 

LFampin exhibits broad antimicrobial action against several gram-positive and gram-negative bacteria, yeasts, and parasites, but no advanced preclinical trials or applications have been reported to date. 

On the other hand, the overall research in this field reveals some bottlenecks such as the synergy between Lfcin-derived peptides and antibiotics, which is relevant, *in vitro* but still far from optimal for *in vivo* therapeutic use [126,127]. Combinations of milk AMPs with a greater number of antimicrobials have to be tested, as they provide new directions to control pathogens growth. Systematic evaluations on *in vivo* models, selecting different pathogens, pathologies and administration routes should be welcomed. Another important issue is the cell-penetrating potential of AMPs as vectors for intracellular targets [128]. 

Because Lfcin and Lfampin are spatially close in location, a generation of chimera peptides containing the same sequences has been produced. Recent studies have shown that chimera peptides exert stronger antimicrobial activity, as well as antiparasitic activity [91,129,130,131]. The construction of chimera peptides, based on the active regions of Lf, may be a promising approach possibly leading to interesting and improved activity. Nevertheless, some shortcoming of such chimera might stem from the difficulty of producing bulk quantities, not fully disclosed toxicity, and stability issues.

Furthermore, besides the molecules object of the present review report, research on different proteolytic fractions of whey proteins (from different mammalian species) lead to continuous discovery of new peptides possessing antimicrobial activities. Hence, the research on such molecules, the evaluation of their spectrum of activities and their *in vitro* and *in vivo* synergy with conventional therapeutics, represent the main goals for the future production of novel peptides.

In conclusion, in recent years, peptides have attracted increasing interest as a new therapeutic approach. They play a key-role due to their wide availability and the broad spectrum of activities, which control many biochemical processes. More than sixty peptide drugs have reached the market, and several hundred new therapeutic peptides are now in preclinical and clinical development. However, the possible ready enzymatic degradation and the low cellular uptake greatly restrict the use of these compounds as pharmaceutical agents. 

A key-factor for enhancing stability and cellular permeability is the application of rational design to partially modify chemical and physical properties of peptides. The challenge is therefore to improve biological and pharmacological activity by making appropriate structural modifications on functional groups present in the amino acids of natural peptides. We believe that in the future, once these transformations will be achieved, the new molecules would benefit of significant stability and cell permeability, which combined with the high efficiency, specificity and security of the natural molecules will ensure success of peptide drugs. 

## Figures and Tables

**Table 1:** Comparison of amino acid sequences of Lf(1–11) in Lf of different mammalian species.

Species	Sequence
Human	GRRRSVQWCAV
Cow	APRKNVRWCTI
Buffalo	APRKNVRWCTI
Horse	APRKSVRWCTI
Goat	APRKNVRWCAI
Camel	ASKKSVRWCTT

For the different amino acids, the colours indicate: blue = positively charged (R, K, H); black = negatively charged (D, E); red = hydrophobic (I, L, V, A, P, M F, Y, W); green = hydrophilic (S, T, E, Q, N, C and G).

**Table 2:** Comparison of aminoacidic sequences of Lfcin from Lf of different mammalian species.

Species	Sequence
Human	TKCFQWQRNMRKVRGPPVSCIKRDS
Cow	FKCRRWQWRMKKLGAPSITCVRRAF
Buffalo	LKCHRWQWRMKKLGAPSITCVRRAF
Horse	AKCAKFQRNMKKVRGPSVSCIRKTS
Goat	SKCYQWQRRMRKLGAPSITCVRRTS
Camel	KKCAQWQRRMKKVRGPSVTCVKKTS
Mouse	EKCLRWQNEMRKVGGPPLSCVKKSS

For the different amino acids, the colours indicate: blue = positively charged (R, K, H); black = negatively charged (D, E); red = hydrophobic (I, L, V, A, P, M F, Y, W); green = hydrophilic (S, T, E, Q, N, C and G).

**Table 3:** Activities of bLfcin against different bacterial, fungal and parasite species. References from [14].

**Gram Positive**	**MIC (µg/mL)**	**Gram Negative**	**MIC (µg/mL)**
*Bacillus subtilis* ATCC 6633	0.6–2	*Escherichia coli* IID-861	6–50
*B. subtilis* 2116	7.8	*E. coli ATCC* 25922	3.3–30
*B. subtilis* IFO-3009	2	*E. coli* O111	6–12
*B. cereus* MMI-272	9	*E. coli* O157:H7-A	8
*B. cereus* 6349	15.6	*E. coli* O157:H7-B	8
*B. circulans* JCM-2504T	0.6	*E. coli* O157:H7-C	10
*B. sphaericus* 7585	1.9	*E. coli* O157:H7-D	8
*Staphylococcus aureus* JCM-2151	6-25	*E. coli* O157 wild type	7.8
*S. aureus* JCM-2179	6	*E. coli* CL99 1–2	4
*S. aureus* JCM-2413	18	*E. coli* K12 UB1005	1.6
*S. aureus* ATCC 25923	30	*E. coli* K12 UB1005 DC-*2*	1.6
*S. aureus* ATCC 29213	6.6	*E. coli* 7275	7.8
*S. aureus* 8530	15.6	*E. coli* 10418	3.9
*S. aureus* 8532	15.6	*E. coli* wild type	15.6–31.2
*S. aureus* R1 (antib-res*)*	12	*Pseudomonas aeruginosa* MMI-603	12–24
*S. epidermidis* JCM-2414T	3–6	*P. aeruginosa* IFO-3445	12–18
*S. epidermidis* 4276	7.8	*P. aeruginosa* IFO-3446	24
*S. haemolyticus* JCM-2416T	1	*P. aeruginosa* IFO-3448	45
*S. hominus* JCM-2419T	3	*P. aeruginosa* IFO-3452	30
*S.* sp. wild type	7.8–15.6	*P. aeruginosa* ATCC-2783	3.3
*Listeria monocytogenes* IDF-Ib	0.6	*P. aeruginosa* PAO	3.3
*L. monocytogenes* JCM-7673	1	*P. aeruginosa* 10662	31.2
*L.* *monocytogenes* JCM-7674	3	*P. aeruginosa* wild type	15.6–31.2
*L. monocytogenes* EGD	1.6	*P. putida* wild type	15.6
*L. monocytogenes* 4b	6.6	*P. cepacia* wild type	250–500
*L. monocytogenes* 5105	1.9	*P. fluorescens* wild type	15.6
*Streptococcus bovis* JCM-5672	3–6	*Pseudomonas fluorescens* IFO-14160	>60
*S. mutans* JCM-5705T	6	*Salmonella enteritidis* IID-604	12–18
*S. mutans* JCM-5175	6	*S. typhimurium* SH7641	1.6
*S. mutans* JCM-5176	3	*S. typhimurium* SL696	5
*S. thermophilus* ATCC-19258	3	*S. typhimurium* 6749	1.6
*S.* *lactis* ATCC-19435	3	*S.montevideo* SL5222	3
*S. cremoris* ATCC-9265	3	*S. newport* 5751	7.8
*Lactobacillus. casei* MMI-114	12	*S. typhi* wild type	7.8–15.6
*Corynebacterium ammoniagenes* JCM-1306	0.3	*S. enteritidis* wild type	7.8
*C. renale* JCM-1322	1	*Yersinia enterocolitica* IID-981	6–24
*C. diphtheriae* JCM-1310	18	*Y. enterocolitica* wild type	62.5
*Clostridium perfringens* ATCC-6013	24	*Proteus vulgaris* JCM 1668T	12–45
*C. paraputrificum* MMI-25	3	*P. vulgaris* wild type	500
*Micrococcus* sp. wild type	7.8–31.2	*P. vulgaris* 4635	500
*Bifidobacterium adolescentis* ATCC-15703	no	*P. mirabilis* wild type	250–500
*B. breve* ATCC-15700	no	*P. mirabilis* NCTC-60	>200
*B. longum* ATCG15707	no	*P. mirabilis* ATCC 35659	>1000
*B. infantis* ATCC-15697	no	*P. rettgeri* wild type	250
*B. bifidum* ATCC-15696	>60 × 10^3^	*P.* sp. wild type	500
*Klebsiella pneumoniae* 418	500	*Klebsiella pneumoniae* JCM 1662T	9
*Enterococcus faecalis* ATCCE 19433	>60	*K. pneumoniae* JCM-16623	12
*Enterococcus* sp. wild type	62.5–125	*K. pneumoniae* 5055	15.6
		*K. pneumoniae* wild type	15.6–62.5
		*K. pneumoniae* 418	500
		*Shigella flexneri* 5452	3.9
		*Shigella flexneri* wild type	3.9
		*Shigella sonnei* wild type	7.8
		*Enterobacter intermedius* wild type	15.6
		*Enterobacter aerogenes* wild type	125
		*Enterobacter cloacae* wild type	70.3
		*Enterobacter* sp. wild type	7.8
		*Serratia* sp. wild type	500
		*S. marcescens* wild type	500
		*S. liquefaciens* wild type	500
		*Citrobacter freundii* wild type	7.8–62.5
		*C. diversus* wild type	62.5
		*Bacteroides distasonis* MMI-M602	no
		*B. vulgatus* MMI-S601	no
**Yeast**	**MIC (µg/mL)**	**Filamentous Fungi**	**MIC (µg/mL)**
*Candida.* *albicans* TIMM 0154	25	*Trichophyton mentagrophytes* TIMM 1189	12
*Candida albicans* TIMM 1768	12.5–400	*T. mentagrophytes* TIMM 2789	6.3
*C. albicans* TIMM 3164	400	*T. mentagrophytes* IFO 5466	30
*C. albicans* TIMM 3315	50	*T. mentagrophytes* IFO 5812	30
*C. albicans* TIMM 3317	200	*T. mentagrophytes* IFO 5974	45
*C. albicans* JCM1542T	24	*T. mentagrophytes*	5
*C. albicans* JCM2072	30	*T. rubrum* IFO 6203	24
*C. albicans* JCM2075	45	*T. rubrum* IFO 32409	13
*C. albicans* JCM2076	24	*T. tonsurans*	5–40
*C. albicans* JCM2374	24	*T. violaceum*	40
*C. albicans* JCM2900	24	*T. rubrum*	>80
*C. albicans* JCM2901	45	*T. shoenleinii*	>80
*C. albicans* JCM2902	60	*Nannizzia incurvata* JCM 1906	18
*C. albicans* JCM2904	45	*N. otae* JCM 1909	60
*C. albicans* 6372	0.8	*N. gypsea* JCM 1903	>60
*C. albicans* 6434	0.8	*Penicillium pinophilum* JCM 5593	45
*C. albicans* ATCC 90028	400	*P. vermiculatum* JCM 5595	45
*C. albicans* wild type	7.8–21.67	*P. notatum*	>80
*C. parapsilosis* wild type	7.8–80	*P. expansum*	>80
*C. tropicalis*	0.31–1.25	*Aspergillus versicolor*	10
*C. glabrata*	120	*A. fumigatus* JCM 1917	>60
*C. guilliermondii*		*A. niger* JCM 5546	>60
*C. kefyr*	2.5–10	*A. fumigatus*	>80
*C. krusei*	10–20	*A. niger*	>80
*Cryptococcus uniguttulatus* JCM 3685	6	*A. flavus*	>80
*C. curvatus* JCM 1532T	9	*A. clavatus*	>80
*C. albidus* JCM 8252	24	*Fusarium moniliforme*	2.5–5
*C. neoformans*	0.63	*Absidia corymbifera*	40
*Trichosporon cutaneum* JCM 2466	18	*Microsporum canis*	40
*T. cutaneum*	1.25–2.5	*M. gypseum*	20–40
*Saccharomyces cerevisiae*	0.63	*Epidermophyton floccosum*	0.31-2.5
		*Fonsecaea pedrosoi*	5
		*Exophiala dermatitidis*	2
		*Phialophora verrucosa*	5–10
		*Cladosporium trichoides*	0.63–1.2
		*Paracoccidioides brasiliensis*	5
		*Sporothrix schenckii*	2–10
		*Rhizopus oryzae* JCM 5557	>60
		*Mucor circinelloides*	>80
		*M. racemosus*	>80
**Parasite**	**LD50 (µM)**		
*Giardia lamblia*	2.8		
*Entamoeba histolytica*	647		
*Toxoplasma gondii*	no		

**Table 4:** Synergistic effects of Lf AMPs [13].

Lf Peptide	Drug	Bacterial, Fungal, Parasite Species	Refs
Lf(1–11)	Fluconazole	*Candida* sp.	[53]
bLfcin	Clotrimazole, ketoconazole, itraconazole, fluconazole	*C. albicans*	[52]
	Cecropin A, aureomycin	*E. coli*	[74]
	Aureomycin	*S. aureus*	[74]
	Fluconazole, itraconazole	*C. albicans*	[84]
	Erythromycin	*E. coli*	[85]
	Ciprofloxacin, ceftazidime, gentamicin	*S. aureus*, *E. coli*	[86]
	Ciprofloxacin, norfloxacin	*E. coli*	[87]
	Minocycline, acid cholic, cysteine, various acylglycerols, β-cyclodextrin	*S. aureus*	[88]
	Metronidazole	*Entamoeba histolytica*	[89]
			
	Nisin, Lf	*E. coli*, *S. epidermidis*	[90]
Lfampin	Ampicillin	*S. aureus*	[91]

**Table 5:** Comparison of amino acid sequences of Lfampin from Lf from six species.

Species	Sequence
Human	WNLLRQAQEKFGKDKSP
Cow	WKLLSKAQEKFGKNKSR
Buffalo	WKLLSKAQEKFGKNKSG
Horse	WKLLHRAQEEFGRNKSS
Goat	WELLRKAQEKFGKNKSQ
Camel	WKLLVKAQEKFGRGKPS

For the different amino acids, the colours indicate: blue = positively charged (R, K, H); black = negatively charged (D, E); red = hydrophobic (I, L, V, A, P, M F, Y, W); green = hydrophilic (S, T, E, Q, N, C and G).

**Table 6:** Activities of Lfampin 265–284 and 268–285 [14].

Gram-Positive	LC_50_ (µM)	Gram-Negative	LC_50_ (µM)	Yeast	LC_50_ (µM)
*Streptococcus sanguis* SK4	4.8	*E. coli* K12	5.8	*Candida albicans* 315 ATCC 10231	0.7–2.1
*Bacillus subtilis* ATCC 9372	5.5–20	*E. coli* O157:H7	25		
*S. aureus* MRSA	20	*E. coli* MREPEC	20	Parasite	
*Streptococcus mutans*	5.5	*E. coli* EPEC E2348/69	20	*Leishmania donovani*	25.3
*Actinomyces naeslundii* ATCC 12104	4.3	*P. aeruginosa* Pak	7		
		*P. aeruginosa* PAO	5.8–15		

## Abbreviations

The following abbreviations are used in this manuscript:

AMPs	antimicrobial peptides
Lf	lactoferrin
Lfcin	lactoferricin
Lfampin	lactoferrampin
MIC	minimal inhibition concentration
LFH	lactoferrin hydrolysate by pepsin
